# A Surgical Kit for Stem Cell-Derived Retinal Pigment Epithelium Transplants: Collection, Transportation, and Subretinal Delivery

**DOI:** 10.3389/fcell.2022.813538

**Published:** 2022-02-18

**Authors:** Kang V. Li, Miguel Flores-Bellver, Silvia Aparicio-Domingo, Carson Petrash, Hannah Cobb, Conan Chen, M. Valeria Canto-Soler, Marc T. Mathias

**Affiliations:** ^1^ CellSight Ocular Stem Cell and Regeneration Research Program, Department of Ophthalmology, Sue Anschutz-Rodgers Eye Center, University of Colorado School of Medicine, Aurora, CO, United States; ^2^ Department of Ophthalmology, Sue Anschutz-Rodgers Eye Center, University of Colorado School of Medicine, Aurora, CO, United States; ^3^ Charles C. Gates Center for Regenerative Medicine, University of Colorado School of Medicine, Aurora, CO, United States

**Keywords:** retinal regeneration, transplantation, surgical instrument, retinal pigment epithelium, human induced pluripotent stem cells

## Abstract

Transplantation of stem cell-derived retinal pigment epithelium (RPE) cells is a promising potential therapy for currently incurable retinal degenerative diseases like advanced dry age-related macular degeneration. In this study, we designed a set of clinically applicable devices for subretinal implantation of RPE grafts, towards the overarching goal of establishing enabling technologies for cell-based therapeutic approaches to regenerate RPE cells. This RPE transplant kit includes a custom-designed trephine for the production of RPE transplants, a carrier for storage and transportation, and a surgical device for subretinal delivery of RPE transplants. Cell viability assay confirmed biocompatibility of the transplant carrier and high preservation of RPE transplants upon storage and transportation. The transplant surgical device combines foldable technology that minimizes incision size, controlled delivery speed, no fluid reflux, curved translucent tip, usability of loading and *in vivo* reloading, and ergonomic handle. Furthermore, the complementary design of the transplant carrier and the delivery device resulted in proper grasping, loading, and orientation of the RPE transplants into the delivery device. Proof-of-concept transplantation studies in a porcine model demonstrated no damage or structural change in RPE transplants during surgical manipulation and subretinal deployment. Post-operative assessment confirmed that RPE transplants were delivered precisely, with no damage to the host retina or choroid, and no significant structural change to the RPE transplants. Our novel surgical kit provides a comprehensive set of tools encompassing RPE graft manufacturing to surgical implantation rendering key enabling technologies for pre-clinical and clinical phases of stem cell-derived RPE regenerative therapies.

## Introduction

Retinal pigment epithelium (RPE) plays an important role in supporting normal photoreceptor function (Strauss, 2005). RPE damage leads to secondary dysfunction and degeneration of photoreceptor cells, which in turn causes severe, irreversible vision impairment in patients affected by conditions such as age-related macular degeneration (AMD) and Stargardt’s disease (Mehat et al., 2018; Flaxel et al., 2020). Recently, clinical trials involving transplantation of embryonic stem cell-derived RPE in patients with AMD showed promising safety and efficacy outcomes (Da Cruz et al., 2018; Kashani et al., 2018; Mehat et al., 2018). Moreover, RPE derived from human-induced pluripotent stem cells (hiPSC), which could be utilized for autologous therapies, is also being evaluated in clinical trials (ClinicalTrials.gov Identifier: NCT04339764; UMIN-CTR number: UMIN000011929) (Mandai et al., 2017).

The above-mentioned clinical trials have used two different cell delivery modalities: an RPE cell suspension or RPE monolayers. When transplanting RPE cell suspensions, commercial injectors are widely used in both pre-clinical and clinical studies (e.g., Hamilton syringe, MedOne PolyTip cannula) (Durlu and Tamai, 1997; Saigo et al., 2004; Carr et al., 2009; Schwartz et al., 2012; Wu et al., 2016; Chao JR et al., 2017; Zhu et al., 2020). In the case of transplantation of RPE monolayers, several groups have developed specific devices customized to fit their needs. Kamao et al. designed a surgical device consisting of a 20G catheter, and a medical 1-ml syringe that can load and eject the RPE sheet through pressure from the plunger by moving a circular metal of the plunger back and forth; the transparent catheter enables RPE sheet delivery under direct visualization (Kamao et al., 2017). Fernandes et al. developed a tissue injector with a lumen and jaws; the jaws allow grasping and loading the RPE graft into the lumen of the injector and minimizes trauma to the graft and host tissue (Fernandes et al., 2017). da Cruz et al. built an apparatus to introduce the RPE patch by advancing a flexible rod through the shaft, while the purpose-built tip protects the RPE monolayer during delivery (Da Cruz et al., 2018). Sharma et al. established a transplantation tool to load and release the RPE transplant utilizing the viscous fluid injector device of the vitrectomy system; the curve of the tool allows the surgeon to deliver the RPE transplants precisely (Sharma et al., 2019). Stanzel et al. invented a shooter instrument for safe delivery of the RPE graft into the subretinal space; by squeezing the actuator, its connected plunger enabled RPE graft loading and delivering conveniently (Stanzel et al., 2012; Liu et al., 2021). In our experience, an optimal subretinal delivery device would minimize incision size within the retina and sclera, provide atraumatic delivery of the RPE graft to the subretinal space, and allow adequate manipulation and visualization of the transplant during delivery. To our knowledge, there is no optimal device that has combined all of these features.

Besides the transplant delivery device, attention should be also drawn to the preservation of the RPE transplants during transportation and surgical manipulation. Previous studies have shown acceptable outcomes in RPE transplants stored for up to 5 and 8 h (Kamao et al., 2017; Da Cruz et al., 2018). A longer preservation time could be helpful for domestic or international express delivery of the RPE transplants.

In this study, we have designed a set of clinically applicable devices to manufacture, transport, and deliver intact hiPSC-derived RPE sheets. This RPE transplant kit includes a custom-designed trephine for the production of RPE transplants, a carrier for storage and transportation, and a surgical device for subretinal delivery of RPE transplants. Cell viability assay confirmed biocompatibility of the transplant carrier and high preservation of RPE transplants upon storage. With our novel surgical transplantation device hiPSC-RPE transplants were delivered in the subretinal space of the pig retina precisely and safely utilizing technology to allow smaller incisions within the retina and sclera. Post-operative evaluation confirmed deployment of the hiPSC-RPE transplants at the target location with no associated damage to the host retina and choroid. Thus, our new devices provide an effective all-encompassing surgical kit for RPE monolayer transplantation.

## Materials and Methods

### Human-Induced Pluripotent Stem Cell-Derived Retinal Pigment Epithelium Cell Culture

A human episomal iPSC line derived from CD34+ cord blood was used in this study (A18945, ThermoFisher Scientific) (Burridge et al., 2011) and routinely tested for *mycoplasma* contamination by PCR. hiPSC culture and retinal organoid differentiation were conducted as previously described (Zhong et al., 2014). hiPSC-derived RPE was generated according to Flores-Bellver et al. (2021). Briefly, retinal organoids at 60 days of differentiation were used to isolate retinal pigment epithelium (RPE) tissue. Dissected RPE spheroids were enzymatically dissociated into single cells and seeded onto Transwell (No.3460, Corning) coated with Matrigel matrix (No.354230, Corning). The transwell membrane is a 10 μm thick polyester membrane of 0.4 μm pore size and 4 × 10^6^/cm^2^ pore density. RPE cells were maintained at 37°C and 5% CO_2_ in MEM-α modified medium (M4526, Sigma), containing 1:100 ml/ml N1 supplement (N6530, Sigma), 250 mg/L taurine (T0625, Sigma), 0.013 μg/L triiodo-l-thyronine (T5516, Sigma), 20 μg/L hydrocortisone (H0396, Sigma), non-essential amino acid solution (M7145, Sigma), and 1:100 ml/ml glutamine-penicillin-streptomycin (G1146, Sigma), and 5% fetal bovine serum (Atlanta Biologicals) as previously described (Maminishkis et al., 2006). Cell culture media was changed every other day and hiPSC-RPE monolayers were used for experiments after 40 days of differentiation.

### hiPSC-RPE Transplant Preparation and Storage Conditions

We used a scalpel to harvest the hiPSC-RPE (with the polyester membrane) from the transwell insert, and the hiPSC-RPE (with the polyester membrane) was transferred to a Petri dish. Then, hiPSC-RPE transplants were obtained from the transwell membranes by means of a custom-designed trephine (MedOne Surgical, FL) as described in results. hiPSC-RPE transplants were then placed in the purpose-built carrier (described in results), and the carrier introduced into a sterile 2 ml tube filled with RPE culture media. The tubes containing the carriers loaded with RPE transplants were divided into 3 groups: one group was maintained within a tissue culture incubator (37°C, 5% CO_2_), a second group was kept at standard room conditions (25°C, 0.04% CO_2_), and a third group was shipped and delivered *via* 24 h express (FedEx, US) (10–23°C, 0.04% CO_2_). A fourth control group was maintained in standard culture conditions in Petri dishes within a cell culture incubator. At the end of the storage period (24 h) viability tests were performed using Ethidium homodimer-1 (EthD-1) (L3224, LIVE/DEATH Cell Viability Assays, ThermoFisher) and Hoechst (H3570, ThermoFisher). Experiments were done in triplicates (3 biological replicates, 2 technical replicates each) including a cell-death positive control induced by treatment with 5% saponin for 10 min at room temperature. Each transplant was imaged as a whole with an inverted microscope (Ti Automated Inverted Microscope, Nikon) and the total number of cells (Hoechst positive) and the number of dead cells (EthD-1 positive) were counted using Imaris 7.0 (Oxford Instruments). To analyze the location of the dead cells within the transplant, transplant areas were defined as follows: 1) edge area corresponding to 20 µm from the edge of the membrane towards the center; 2) central area corresponding to the remaining surface of the transplant.

### Animals

Wild type domestic pigs (n = 2; 6 months old, females; National Swine Resource and Research Center, University of Missouri, MO) were used to evaluate the performance of our new RPE transplant delivery device. The animal study was approved by the University of Colorado Institutional Animal Care and Use Committee and carried out in strict accordance with the Association for Research in Vision and Ophthalmology (ARVO) Statement for the Use of Animals in Ophthalmic and Vision Research and the ARRIVE guidelines (Percie Du Sert et al., 2020).

### hiPSC-RPE Transplantation Device and Surgical Procedure

Pigs were sedated with Telazol 10 mg/kg (Fort Dodge Animal Health, IA), and anesthesia was maintained on isoflurane (1.5–2.5%). Heart rate, respiration and temperature were continuously monitored. The periocular area was sterilized with 5% povidone-iodine solution. Phenylephrine Hydrochloride Ophthalmic Solution (2.5%) and Tropicamide Ophthalmic Solution (1%) were used for pupil dilation. A lid speculum was placed and 2% lidocaine injected sub-tenon. Localized conjunctival peritomies were performed to expose sclera and 3 surgical ports were placed 3.5 mm from the limbus using 23 gauge valved trocar cannulas (Alcon Surgical). A core vitrectomy was performed (Constellation, Alcon Surgical). Intravitreal triamcinolone was injected into the vitreous cavity to stain the posterior hyaloid. Using aspiration, a posterior vitreous detachment was induced and the peripheral vitreous shaved. Subsequently, subretinal surgical procedures were adapted from Koss et al. (2016) and Fernandes et al. (2017). Briefly, a localized retinal bleb was created in the superonasal visual streak using a PolyTip Cannula 25/38G (MedOne Surgical). Endodiathermy was applied to the base of the retinal bleb for hemostasis and a retinotomy was created using intraocular scissors. hiPSC-RPE transplants (carrier-incubator group) were loaded into the transplantation device from the transplant carrier; the tip of the transplantation device was introduced into the vitreous cavity through a separate pars plana scleral incision and the hiPSC-RPE transplants were released into the subretinal space. After transplant delivery, the sclerotomy was sutured immediately to stabilize intraocular fluid dynamics. Fluid air exchange and laser photocoagulation were performed to flatten the detached bleb and seal the retinotomy. The sclerotomies and conjunctival incisions were closed with a 7–0 absorbable suture (Vicryl, Ethicon). Neomycin and Polymyxin B Sulfates, Bacitracin Zinc and Hydrocortisone Ophthalmic Ointment (Bausch & Lomb) was placed in the eye, and the eye patched and shielded. Pigs were subjected to immunosuppression regime based on suprachoroidal injection of triamcinolone acetonide (4 mg; Amneal Pharmaceuticals) 1 week before surgery, intramuscular injection of methylprednisolone acetate (5 mg/kg; Teva Pharmaceuticals) and sub-tenon injection of triamcinolone acetonide (20 mg; Amneal Pharmaceuticals) at the time of surgery, followed by oral prednisone (1 mg/kg; Par Pharmaceutical) daily until end of study.

### Evaluation After Transplantation Surgery

*In vivo* assessment was performed by spectral domain optical coherence tomography (SD-OCT) (Envisu, Bioptigen) 1 week post transplantation surgery. Histological and immunohistochemical analysis was also performed. Enucleated eyes were cryopreserved according to previously published protocols (Cuenca et al., 2018). The tenon’s capsule and extraocular muscles were removed as much as possible, and a cut was made around the limbus with a scalpel blade to allow the fixative solution to enter into the eye cavity. Eyes were fixed in 4% paraformaldehyde for 2 h at room temperature. After washing with 0.1 M sodium phosphate buffer (pH 7.4), eyes were immersed in increasing concentrations of sucrose (up to 25%) before freezing. Cryosections (12 mm thickness) were collected and processed for immunofluorescent labeling with anti-Ku80 antibody (rabbit anti-human, 1:200, ab80592, Abcam), and melanosome antibody (mouse anti-human, 1:200, HMB45, Dako). DAPI (4′,6-diamidino-2-phenylindole) was used for nuclear counterstaining (Molecular Probes). Fluorescence images were acquired with a confocal microscope (C2, Nikon).

### Statistics and Graphs

Statistical analysis was performed using one-way ANOVA in SPSS Statistics Version 23.0 (SPSS Inc., IBM Company, Armonk, NY) and Prism Software Version 7 (GraphPad Software Inc., La Jolla, CA). The differences were considered statistically significant when the *p*-value was less than 0.05. The cartoons in Figures 2A, 3A were created with BioRender.com (agreement number: RG22SB5FYK, QD22SB5ZJ4).

## Results

### Generation of Human-Induced Pluripotent Stem Cell-Derived Retinal Pigment Epithelium

**FIGURE 1:**
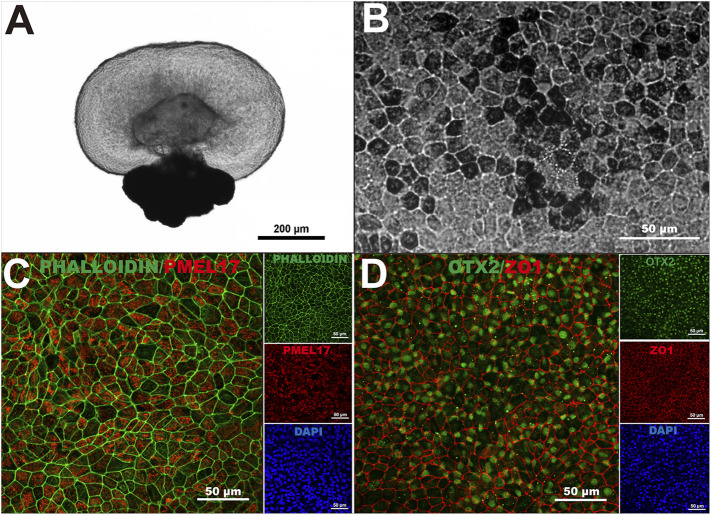
Generation of human-induced pluripotent stem cell-derived retinal pigment epithelium. **(A)** A three-dimensional retinal organoid containing neural retina and retinal pigment epithelium (RPE) cells representative of the ones used in this study. **(B)** hiPSC-derived RPE showed characteristic pigmentation and typical cobblestone morphology upon 40 days in culture. **(C)** Intact F-actin cytoskeleton (Phalloidin) and proper localization of premelanosome protein (PMEL17) in hiPSC-derived RPE monolayers at 40 days. **(D)** Orthodenticle homeobox 2 (OTX2), and zonula occludens-1 (ZO-1) also showed proper pattern of expression in hiPSC-derived RPE monolayers at 40 days in culture. n > 3.

We previously demonstrated that human-induced pluripotent stem cells (hiPSC) can form three-dimensional retinal organoids containing neural retina and retinal pigment epithelium (RPE) cells *in vitro* (Zhong et al., 2014; Flores-Bellver et al., 2021). To generate hiPSC-derived RPE monolayers to use as substrate for our RPE transplants, we harvested the RPE spheroids from retinal organoids (Figure 1A), and cultured the dissociated RPE cells on 10 μm thick transparent polyester membranes. After 40 days of differentiation, hiPSC-RPE cells showed the characteristic pigmentation, typical hexagonal morphology, and intact F-actin cytoskeleton (Figures 1B,C). We next characterized our hiPSC-RPE cells by the expression of key proteins involved in normal RPE cell differentiation and function, including premelanosome protein (PMEL17), orthodenticle homeobox 2 (OTX2), and zonula occludens-1 (ZO-1). While PMEL17 is known to be enriched in premelanosomes (Lee et al., 1996; Raposo et al., 2001), OTX2 is crucial for differentiation of RPE cells and transactivation of the genes involved in melanosome formation (Martinez-Morales et al., 2001; Martinez-Morales et al., 2003), and ZO-1 is a membrane-associated tight junction adaptor protein that links junctional membrane proteins to the cytoskeleton and plays an important role in RPE homeostasis *in vivo* (Paris et al., 2008; Georgiadis et al., 2010). The appropriate expression of PMEL17, OTX2, and ZO-1 confirmed that our hiPSC-RPE tissue achieved a healthy and functionally mature state (Figures 1C,D).

### Production of hiPSC-RPE Transplants

**FIGURE 2:**
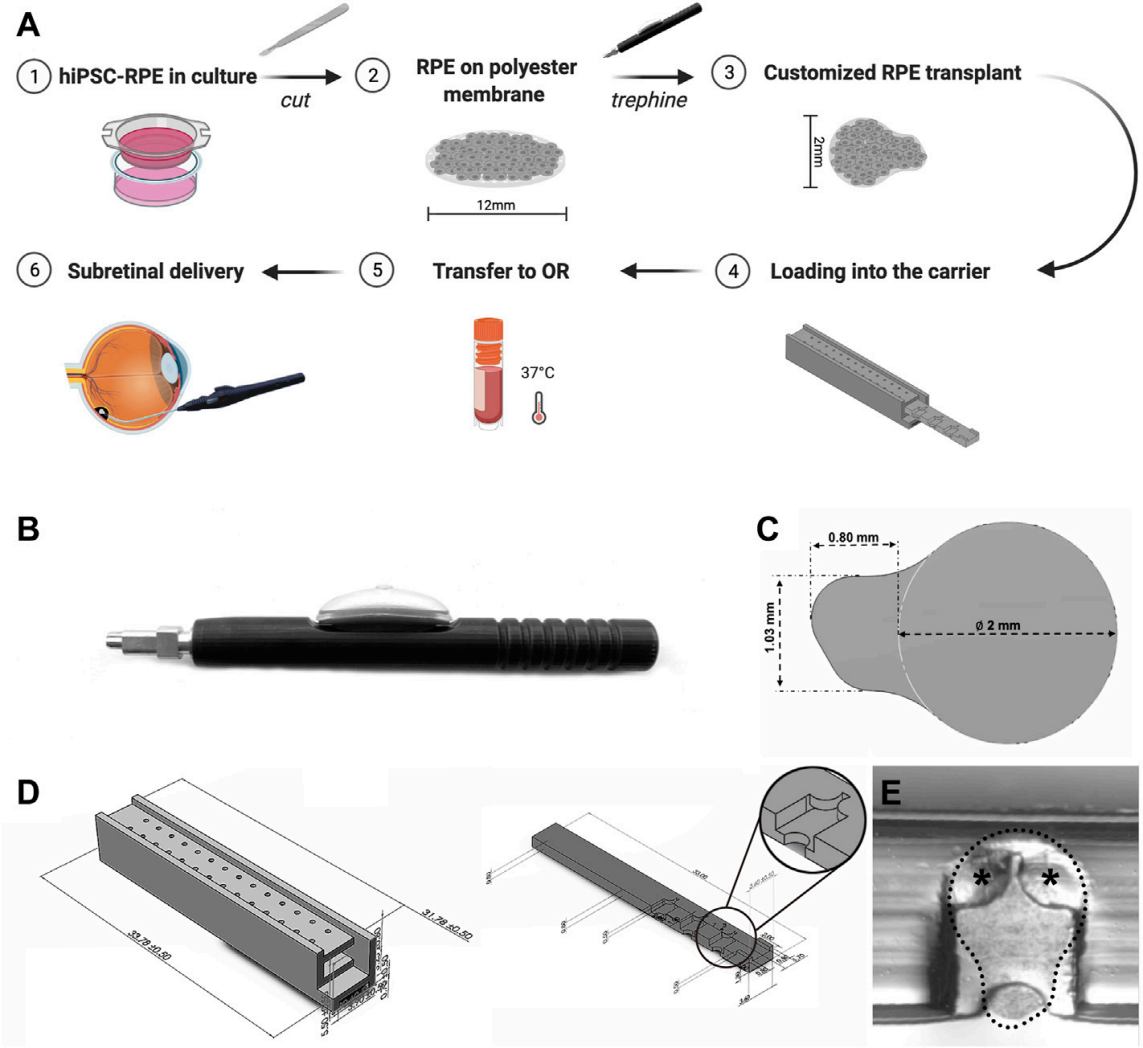
Custom-designed trephine and carrier for production and preservation of hiPSC-RPE transplants. **(A)** Brief workflow outlining production, preservation, transportation, and sub-retinal delivery of hiPSC-RPE transplants and the custom-designed devices developed for each step. **(B)** Custom-designed trephine for hiPSC-RPE transplant production. **(C)** Shape and size of the hiPSC-RPE transplants produced with the trephine. **(D)** The transplant carrier includes a chest (left), and a drawer (right); the drawer contains chambers for docking the hiPSC-RPE transplants, and by sliding into the chest, the drawer protects the transplants from moving or damage. **(E)** A hiPSC-RPE transplant positioned inside a chamber of the drawer (dotted line); two small symmetrical flat pieces at the top of the chamber (asterisks) prevent the hiPSC-RPE transplants from floating away from the chamber.

To obtain hiPSC-RPE transplants, we used a custom-designed trephine according to the desired size and shape of the transplants (Figures 2A–C). The RPE graft was designed as a circular scaffold surface 2 mm in diameter to target atrophic RPE lesions within the clinical macula of AMD patients (Spitznas, 1975; Treumer et al., 2011). At one edge of the circular graft surface, we designed an asymmetric tab (1.03 mm × 0.80 mm) to facilitate transplant manipulation and loading. By grasping at the tab of the transplant with our surgical transplantation device, the RPE transplant slides into the lumen of the transplantation device without damage to the RPE graft itself. Furthermore, the asymmetric design of the tab allows the surgeon to easily identify the proper orientation of the RPE transplant by identifying the side of the scaffold that is covered by the RPE monolayer from the non-cellular side.

### Preservation and Transportation of hiPSC-RPE Transplants

A critical stage of the transplantation process is transportation and viability preservation during transport of the hiPSC-RPE transplants. To address this important need we set to develop a transplant carrier designed to safely transport the hiPSC-RPE transplants, ensuring proper orientation of the transplant, and avoiding preservation media leakage, contamination, and any possible mechanical damage to the transplants. The hiPSC-RPE transplant carrier was made by three-dimensional (3D) printing using VeroClear. VeroClear was selected as the 3D printing material because its optical clarity enables the visualization of RPE transplants stored inside. The carrier is composed of two separate components: a drawer and a chest (Figures 2A,D). The drawer is rectangular in shape (33.00 mm × 3.60 mm × 0.80 mm) and has symmetrically distributed chambers (capable of accommodating a maximum of 7 chambers) for hiPSC-RPE transplants placement. The size of each chamber is 3.20 mm × 2.00 mm × 0.50 mm, matching the size of our hiPSC-RPE transplants. Two small symmetrical flat pieces at the top of the chamber act as a “roof” and prevent the hiPSC-RPE transplants from floating away from the chamber (Figure 2E). The chest component is a 33.78 mm × 3.90 mm × 5.50 mm cuboid. Two rows of through holes enable free flow of the preservation medium within the chest. The drawer containing the hiPSC-RPE transplants slides into the hollow center of the chest smoothly. The whole carrier can be perfectly set into a 2 ml tube without wiggling during transportation.

**FIGURE 3:**
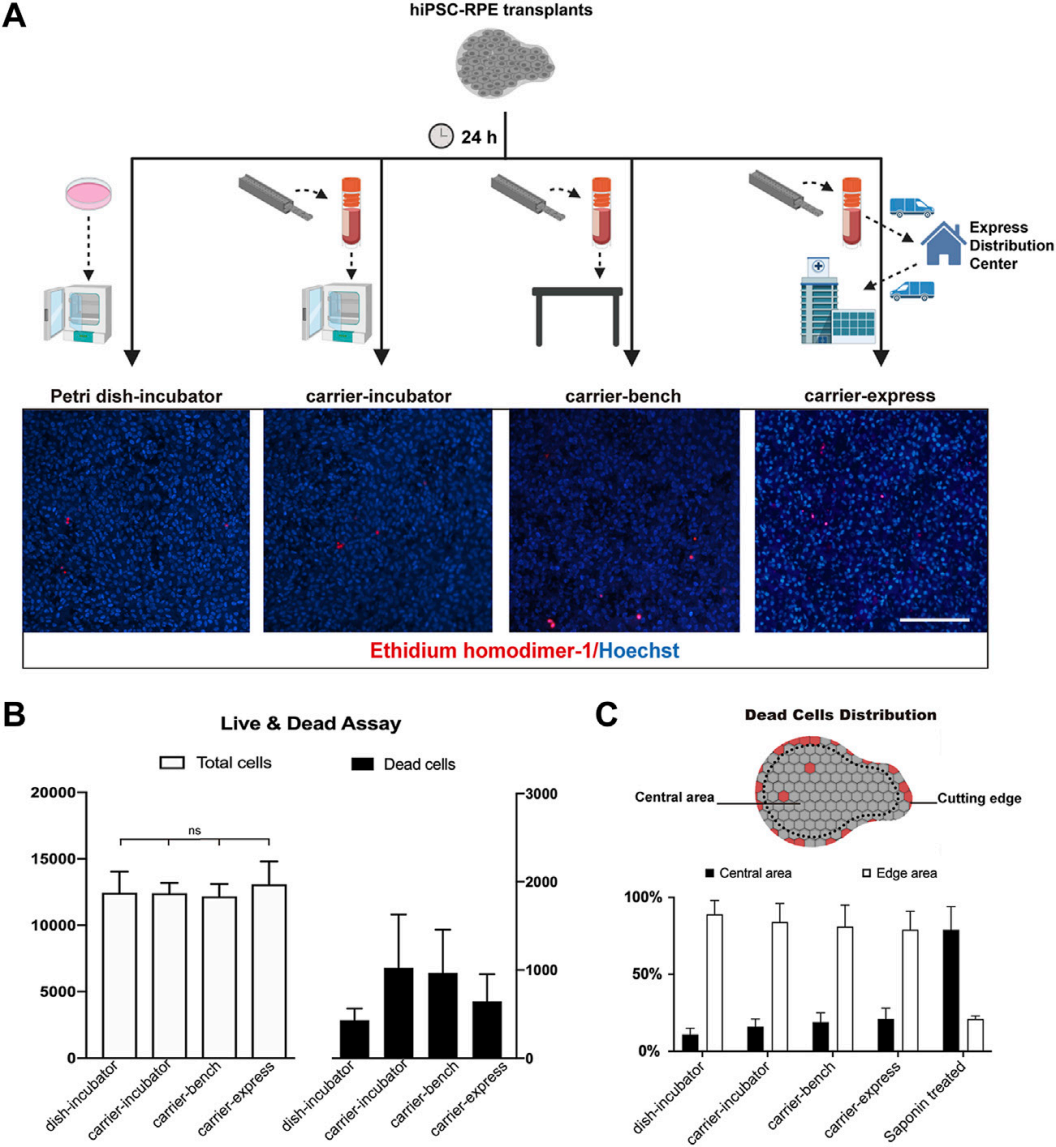
Cell viability of hiPSC-RPE transplants stored in the carrier. **(A)** Workflow and representative pictures of live and dead cell viability assay in hiPSC-RPE transplants incubated for 24 h in Petri dish in a cell incubator (37°C, 5% CO_2_; Petri dish-incubator group; control), carrier in a cell incubator (37°C, 5% CO_2_; carrier-incubator group), at standard room conditions (25°C, 0.04% CO_2_; carrier-bench group), and transported from the lab to the hospital by express shipping (10–23°C, 0.04% CO_2_; carrier-express group). **(B)** Total cell number (white bars) and dead cell number (dark bars) in carrier-incubator, carrier-bench, carrier-express and Petri dish-incubator groups. **(C)** Distribution of dead cells within hiPSC-RPE transplants among carrier-incubator group, carrier-bench group, carrier-express group, Petri dish-incubator group, and saponin-treated group.

In previous studies, Kamao et al. demonstrated that the stem cell-derived RPE graft viability in graft storage medium could be maintained up to 5 h after graft preparation (Kamao et al., 2017). da Cruz et al. preserved stem cell-derived RPE transplants in saline up to 8 h before transplantation (Da Cruz et al., 2018). Here we evaluated the biocompatibility and performance of our transplant carrier on hiPSC-RPE transplant preservation upon 24 h storage time (Figure 3A). hiPSC-RPE transplants were incubated for 24 h in the presence of RPE culture media (preservation media) under the following conditions: 1) transplant carrier in a cell incubator (37°C, 5% CO_2_; carrier-incubator group); 2) transplant carrier at standard room conditions (25°C, 0.04% CO_2_; carrier-bench group); 3) transplant carrier using standard 24 hours express (10–23°C, 0.04% CO_2_; carrier-express group); 4) Petri dish in a cell incubator (37°C, 5% CO_2_; dish-incubator group; control); and 5) Petri dish in a cell incubator treated with saponin (saponin-treated group; cell death positive control). At the end of the 24 h storage time, using the Live/Death Cell Viability Assay (ThermoFisher) we determined that the total cell number of the hiPSC-RPE transplants in the dish-incubator, carrier-incubator, carrier-bench, and carrier-express groups were 12,435 ± 1,596, 12,400 ± 786, 12,166 ± 932, and 13,075 ± 1722 respectively, with no significant differences among the four groups while the total cell number of the saponin-treated group was significantly lower (567 ± 3,953, *p* < 0.05). More importantly, we determined no significant differences in the number of dead cells among the carrier-incubator group (8.26%), the carrier-bench group (7.95%), and the carrier-express group (6.82%). Furthermore, the number of dead cells in these three groups showed no significant differences compared to the dish-incubator (control) group (3.47%, *p* > 0.05) (Figure 3B). Based on these results we infer that VeroClear itself is nontoxic to the RPE cells and therefore a suitable biocompatible material for our transplant carrier. We next asked whether the observed cell death was caused by the environmental conditions within the carrier or by mechanical injury caused by the trephine. To address this, we analyzed the distribution of dead cells and found that most of the dead cells were at the periphery of the transplant outlining the trephine-cutting edge (dish-incubator group 89%, carrier-incubator group 84%, carrier-bench group 81%), carrier-express group 79% (Figure 3C), indicating that most of cell death resulted from the trephine, and that the carrier provided a suitable environment for preserving hiPSC-RPE transplants viability, even in express shipment conditions.

### hiPSC-RPE Transplant Delivery Device

**FIGURE 4:**
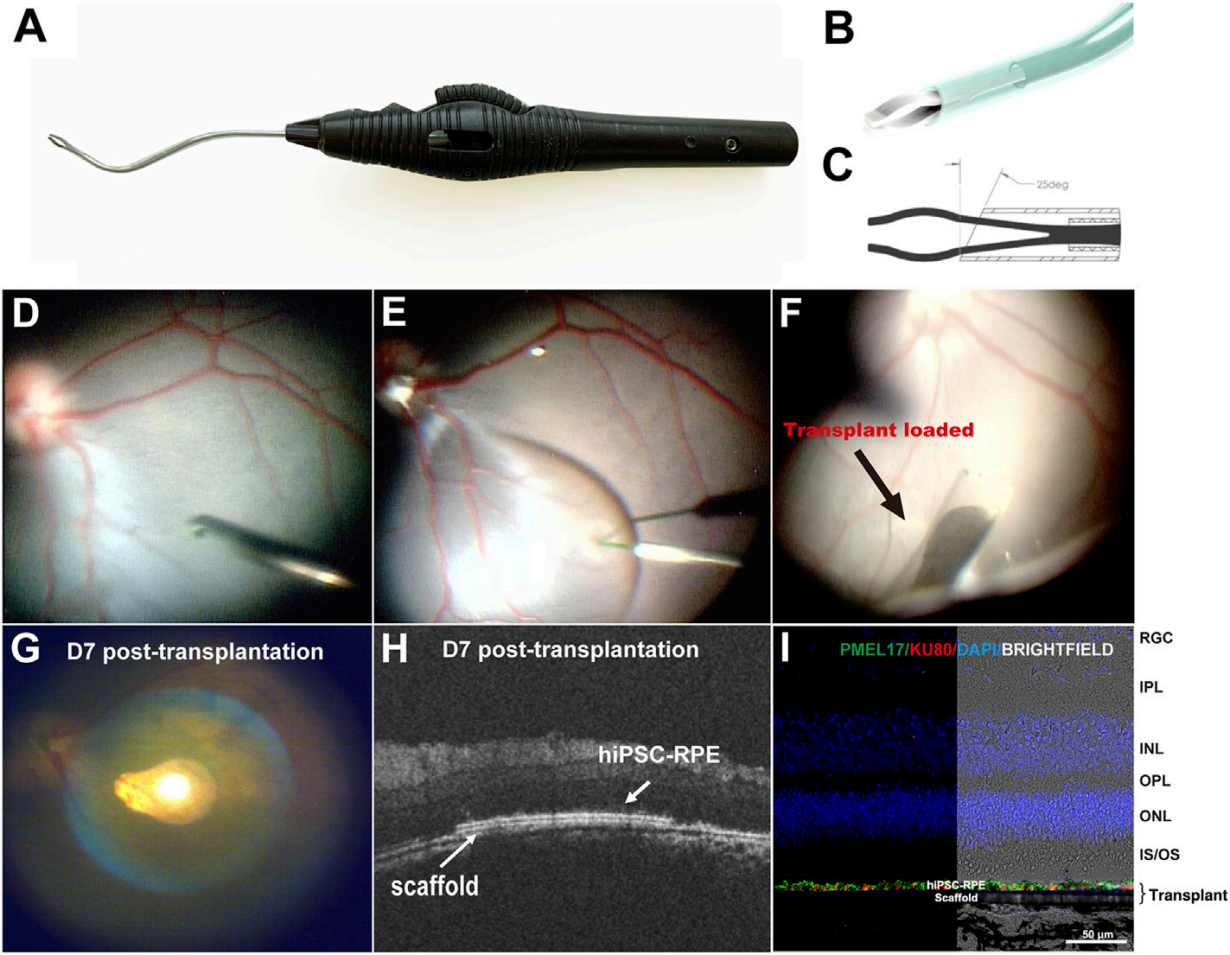
Surgical delivery instrument and outcome of subretinal delivery of the hiPSC-RPE transplants. **(A)** Lateral view of the transplantation device. **(B)** The bent translucent Teflon cannula enables the visualization of the transplant during loading and releasing. **(C)** A 25-degree tip allows the transplant to roll concavely into the lumen of the Teflon cannula. **(D)** During the transplantation surgery, a standard pars plana vitrectomy was performed, **(E)** A localized retinal bleb was created using a Polytip Cannula 25/38G, and **(F)** hiPSC-RPE transplant was delivered within the subretinal space with our surgical delivery instrument. **(G–I)** A week after transplantation surgery, **(G)** fundus photo showed the hiPSC-RPE transplant properly localized at the target position; **(H)** OCT demonstrated that the transplant laid flat between the host neural retina and RPE with no undesired injuries observed in the host retina, RPE and choroid; **(I)** Immunofluorescence for detection of human PMEL17 and human nuclear Ku80 proteins showed a continuous human RPE monolayer covering the polyester membrane scaffold; no human cells were found outside the scaffold. RGC, retinal ganglion cell; IPL, inner plexiform layer; INL, inner nuclear layer; OPL, outer plexiform layer; ONL, outer nuclear layer; IS/OS, inner segments/outer segments; hiPSC-RPE, human induced pluripotent stem cell-derived retinal pigment epithelial cells.

A hiPSC-RPE transplant surgical device was designed to deliver the transplant into the subretinal space (Figures 4A–C). The device consists of an 88-mm-long ergonomic handpiece and an 18-gauge curved shaft with micro-jaws. The handpiece has a wheel that can be turned to advance and retract the tubular shaft. By rolling the wheel forward, the shaft moves forward and eventually slides over the jaws. As the shaft slides over the jaws, it forces them to close, enabling them to grasp the tab of the RPE transplant. As the shaft continues to advance past the jaws, the transplant folds concavely into the lumen, protecting the transplant during surgical manipulation and delivery, and minimizing the size of the scleral and retinal incisions. When the instrument tip is positioned at the delivery location, the transplant can be delivered using the reverse process. By rolling the wheel backward, the shaft retracts and the transplant unfolds back to its initial shape and the jaws re-open releasing the transplant into the target location. The curved translucent Teflon shaft fits the curve of the posterior pole of the retina allowing a better approach angle to the delivery location and enables the visualization of the transplant during loading and releasing thus making the subretinal delivery precise.

To evaluate the safety and reproducibility of the surgery procedures, we delivered four hiPSC-RPE transplants into two WT pigs (one transplant per eye). At first, we performed standard 3-port pars plana vitrectomy (PPV). The ports were placed 3.5–4 mm from limbus to prevent injury to the lens because the pig’s lens thickness (about 7 mm) is thicker than human’s (about 4 mm) (Wong et al., 2007; Jonas et al., 2012). We then created a retinal bleb at the visual streak in the superonasal quadrant of the retina. This specific area was chosen because the visual streak has the highest cone density resembling the human macula, and the superonasal quadrant has relatively fewer vessels, thus minimizing hemorrhage during and post-operation (Chandler et al., 1999). A 1.5 mm incision was made at the base of the bleb, and the tip of the cannula (containing the hiPSC-RPE transplant) was inserted subretinally through the incision. Through the translucent tip of the cannula, hiPSC-RPE transplants were released under wide field visualization to the target position accurately in all operated eyes without causing any iatrogenic injuries such as host RPE detachment, retinal damage, or choroidal hemorrhage (Figures 4D–F). Supplementary Video S1 highlights the complementary design of the transplant carrier and delivery device and the ease of workflow during surgical procedure.

### Post-Transplantation Surgery Evaluations

Spectral Domain Optical coherence tomography (SD-OCT) is an imaging technique that can provide *in vivo* morphological evaluation of the transplants after surgery (Li et al., 2017; Liu et al., 2020). Thus, using OCT we assessed the outcome of the hiPSC-RPE graft transplantation 1 week post-surgery and found that all the transplants were located stably at the target position, with no retinal detachment, hemorrhage or any other visible damage to the host tissues (Figures 4G,H). Furthermore, in enucleated eyes immunofluorescence detection of the anti-human nuclear antigen proved that the hiPSC-RPE cells were attached to the polyester membrane forming a continuous monolayer under the host neural retina (Figure 4I). No human cells were found outside the polyester membrane suggesting that there was no significant hiPSC-RPE cell detachment from the polyester membrane during the delivery process.

## Discussion

The transplantation surgical kit reported here addresses the needs for all steps involved in hiPSC-RPE transplantation process including the generation of asymmetric RPE transplants using a trephine, transportation and preservation of RPE transplants before surgery by use of the carrier, and safe and accurate subretinal delivery of hiPSC-RPE transplants with the subretinal delivery device. Our tools offer a safe and effective all-encompassing surgical kit for RPE monolayer transplantation.

In recent years, increasing attention has been given to RPE monolayer transplantation as an improved strategy for RPE regenerative therapies compared to RPE single cell suspensions (Kamao et al., 2017; Kashani et al., 2018; Mehat et al., 2018; Sharma et al., 2019). For this approach to be successful, delivery of the RPE monolayer with the proper orientation is critical to ensure appropriate function of RPE to maintain the health and integrity of photoreceptors (Bharti et al., 2006; Bharti et al., 2011). Various asymmetrical transplants have been previously designed to facilitate delivery of the RPE monolayers with the proper orientation (Fernandes et al., 2017; Kamao et al., 2017; Da Cruz et al., 2018). What is more, the addition of a tab to the graft is helpful to facilitate grabbing, transferring and loading. Accordingly, we designed a trephine with a customized shape including a circular transplant area and an asymmetrical tab that allows for easy identification of the surface covered by the RPE monolayer, and convenience for loading the RPE transplants into the transplantation device.

Another critical aspect for the success of RPE transplantation is ensuring tissue preservation during storage and transportation time. In previous studies stem cell-derived RPE transplants were stored for up to 8 h in Petri dishes or tubes prior to transplantation surgery (Kamao et al., 2017; Da Cruz et al., 2018). Compared to Petri dishes, sealed tubes are easier to transport and less prone to contamination and leakage. Nonetheless, neither of these methods provide a proven safe, reproducible, and reliable system for ensuring long-term viability and ease of transport of RPE transplants. The RPE transplant carrier reported herein addresses this important need. Our carrier perfectly fit the size of the commercial 2 ml tubes to prevent movement during transportation. Importantly, RPE transplants stored at 25°C, 0.04% CO_2_ as well as those shipped on express courier, showed comparable viability to the ones stored in 37°C, 5% CO_2_. We therefore anticipate that the RPE transplants might tolerate mild environment changes, at least in a relatively short time (24 h) and be amenable to controlled shipment conditions.

Several RPE transplantation devices have been previously designed, all of them being highly customized to fit the various shapes and sizes of the RPE transplants. In all cases, these devices provide some advantageous properties while lacking other significantly important features. Minimization of surgical trauma and usability are highly desirable properties to ensure high performance of transplant delivery instruments. Elements affecting surgical trauma include injection speed control, shape and orientation of the device tip, and size of sclerotomy and retinotomy. In our preliminary studies (data not shown), the straight shaft of the transplantation device could easily lead to trauma in the host RPE or Bruch’s membrane during subretinal delivery due to the steep angle required by a straight shaft approaching a curved surface. Thus, a curved tip that fits the curvature of the posterior pole of the eyeball was incorporated in our final design and is preferred and recommended (Kamao et al., 2017; Da Cruz et al., 2018; Sharma et al., 2019). The ability to control delivery speed is also essential for minimizing trauma in such delicate surgery. This can be accomplished with the use of a wheel gear, a common feature in many surgical devices. Use of the viscous fluid injector device of the vitrectomy system has been used as an alternative option, however this results in additional fluid being delivered into the bleb along with the implant which may cause other difficulties such as implant drift in the stream (Kamao et al., 2017; Kashani et al., 2018; Mehat et al., 2018; Sharma et al., 2019). Finally, a foldable transplant enables a smaller incision in both scleral and retina. With the foldable design, Fernandes et al., 2017 delivered a 3.5 mm wide RPE transplant with a 1.5 mm scleral incision. Another advantage of the foldable design is that the RPE is protected within the lumen of the device avoiding scratching during the loading and delivery process (Luo et al., 2018). As mentioned before, usability is another highly desirable property. Elements affecting usability include loading procedure, ability to reload *in vivo*, and ergonomic handle. For example, transplantation devices featuring forceps or injectors permit a one-step direct loading of the RPE transplants *in vitro* which is convenient for the surgeon. Moreover, a rare but possible scenario is when unexpected intraoperative complications happen, and the RPE transplant needs to be reloaded in the device for delivering to the target area or removing it from the vitreous cavity; once again a forceps-based design like the one in our delivery instrument enables re-grasping of the transplants *in vivo*. Finally, an ergonomic handle also improves the usability of the device because it not only enhances the comfort of surgeons performing the procedure but also improves efficiency when completing the tasks (Sancibrian et al., 2014). The transplantation device we developed combines all these essential properties and is therefore the first to provide foldable technology that minimizes incision size, controlled delivery speed, no fluid reflux, curved translucent tip, usability of loading and *in vivo* reloading, and ergonomic handle.

Worth noting, our surgical kit is highly customizable and could be adapted to fit specifications for a variety of retinal grafts, including RPE grafts of different shapes and sizes, and eventually even stem cell-derived retinal sheets and composite transplants containing RPE and neural retina. These could be achieved by minor adjustments to the design of our trephine, carrier, and delivery instrument. In addition, our carrier can be also adapted by incorporating a temperature- and CO_2_-controlled system to enable global transportation of transplants even in harsh climate.

## Conclusion

This study provides a practical surgical kit for generation, preservation, and subretinal delivery of RPE transplants in pre-clinical studies. Furthermore, all devices included in our kit can be deployed into clinical settings for clinical trials and eventually standard of care for diseases requiring regeneration of RPE tissue.

## Data Availability

The original contributions presented in the study are included in the article/Supplementary Material, further inquiries can be directed to the corresponding authors.

## Supplementary Material

Supplementary Video S1 Video animation showing the complementary design of the transplant carrier and delivery device and the ease of workflow during surgical procedure.
